# Assembled Cantilever Fiber Touch Trigger Probe for Three-Dimensional Measurement of Microstructures

**DOI:** 10.3390/s17112652

**Published:** 2017-11-20

**Authors:** Limin Zou, He Ni, Peng Zhang, Xuemei Ding

**Affiliations:** Department of Automatic Test and Control, Harbin Institute of Technology, Harbin 150080, China; nihe@hit.edu.cn (H.N.); zper@stu.hit.edu.cn (P.Z.); xmding@hit.edu.cn (X.D.)

**Keywords:** fiber optics probe, touch trigger probe, three-dimensional metrology, micro/nano coordinate measurement machine (CMM)

## Abstract

In this paper, an assembled cantilever fiber touch trigger probe was developed for three-dimensional measurements of clear microstructures. The probe consists of a shaft assembled vertically to an optical fiber cantilever and a probing sphere located at the free end of the shaft. The laser is emitted from the free end of the fiber cantilever and converges on the photosensitive surface of the camera through the lens. The position shift of the light spot centroid was used to detect the performance of the optical fiber cantilever, which changed dramatically when the probing sphere touched the objects being measured. Experimental results indicated that the sensing system has sensitivities of 3.32 pixels/μm, 1.35 pixels/μm, and 7.38 pixels/μm in the x, y, and z directions, respectively, and resolutions of 10 nm, 30 nm, and 5 nm were achieved in the x, y, and z, respectively. An experiment on micro slit measurement was performed to verify the high aspect ratio measurement capability of the assembled cantilever fiber (ACF) probe and to calibrate the effective two-point diameter of the probing sphere. The two-point probe sphere diameter was found to be 174.634 μm with a standard uncertainly of 0.045 μm.

## 1. Introduction

With the rapid development of the semiconductor industry, precision engineering industry, micro-system technology, and materials science, many microstructures must be measured with higher precision. The size of these nano- and microstructures ranges from nanometers to hundreds of micrometers [1]. Significant measurement problems arise because the existing surface and coordinate measuring techniques are not fully suitable for the measurement of microstructures [2,3]. The coordinate measurement machine (CMM) is composed of two main parts: the precision positioning machine and the probe. The current positioning machine has achieved sub-nano positioning resolution in the range of several tens of millimeters [4]. Therefore, the main challenge in microstructure measurement is the development of micro-CMM probes with nanoscale measurement resolution and three-dimensional (3D) measurement capability.

The tactile probing system has been widely used for the measurement of microstructures [5,6]. In 1999, National Physical Laboratory (NPL) developed a small probe that had a light structure [7]. The sensors consist of a floating plane, sensitive beams, a stylus, and a probe tip. The floating plane is suspended by the cross-shape sensitive beams, and the stylus and probe tip are fabricated on the floating plane. The deflections of the stylus and probing sphere are transformed into voltage by capacitive sensors on the sensitive beams. Combined with Micro-Electro-Mechanical System (MEMS) machining techniques, some compact probes have been developed based on the floating plane structure [8,9,10]. The tactile probe can help achieve high-sensitivity 3D measurement. The probe developed by METAS [11,12], based on a parallel kinematic structure of flexure hinges, has a resolution of less than 1 nm. The translational motion is separated into its XYZ components, measured by three inductive sensors. However, the probing systems generate large contact probing force, and may cause plastic deformation damage during the probing process. To reduce the contact probing force, the probing system should be adequately flexible. A novel tactile probe with variable stiffness was developed by Kinnell et al. [13]. However, a practical limitation of the probing system is the length of time required, at 20 s, to reach a steady state when the controller switches to flexible mode. 

Some vibrating micro-CMM probes with minimal contact force have been developed. A micro-CMM probe is driven by six piezoelectric (PZT) actuators distributed on three suspending arms [14]. Intersection with the measurement surface produces a vibration amplitude change that can be detected by sensor PZTs. Though the performance of this probing system is satisfactory, fabricating the stylus is difficult. The probe manufacturing process is intricate and requires several rounds of electroplating, lithography, and high assembly accuracy. If the micro probe is not precisely attached to the center of the floating plate, the dissymmetrical structure will result in output non-linearity. The vibration can also be produced by a quartz tuning fork [15,16]. The optical fiber, about 1 mm long, is mounted on the sensing arm as the probe tip [15]. The resonance parameters of the quartz tuning fork are extremely sensitive to external forces. The probe has achieved sub-nanometer resolution in three directions. Since the probe is working in a vibrating state, the length of the probe should not be too long to ensure that the probe can vibrate stably. So, the length of the fiber limits its measurement range along the z-axis and it has difficultly measuring a high aspect ratio microstructure. A 700:1 high aspect ratio probe made by carbon fiber with diameter of 7 μm was developed [16]. However, the probe shanks need to be carefully aligned with the direction of oscillation of the tuning fork to achieve a planar oscillatory motion, making the probe system set up difficult.

Some micro-CMM probes with high precision, based on reflected beam detection, have been applied to 3D measurement [17,18,19]. A reflective plane is fabricated at the end of the probe stylus. The tilt measuring system and the interferometer are used to detect the deflection of the mirror and thus of the probe stylus. The detection method, based on detecting reflected light, can achieve high nanoscale measurement resolution, but manufacturing a reflected membrane on a small probe is challenging. Additionally, the entire system is complicated, and the optical path calibration is difficult. Dai et al. developed an ultra-precision reflected light detection method based on an atom force microscope (AFM). The AFM achieves 3D measurement with a shaft fabricated on the AFM cantilever beam [20,21]. The detection principle of the micro-CMM probe system is the same as a commercial AFM, meaning both have the same measurement resolution. The advantage of the assembled cantilever structure is that its detection optical path is located above the objects to be measured, and the structures of the objects have little effect on measurement. Although the probing system has an excellent measurement performance, the measurement range for depth is still limited by the length of the shaft, which is 0.2 to 2 mm.

Optical fiber has been widely applied in the field of microstructure measurement as its advantages include small measuring force, low cost, and easy processing. Some high-precision fiber probes have already been commercialized [22]. In 2001, Physikalisch-Technische Bundesanstalt (PTB) developed an opto-tactile sensor [23]. However, due to the shadowing effect, this probe cannot detect high aspect ratio structures. Based on the probe, a fiber probe that can measure the inner-dimensions of microstructures was developed [24]. To obtain higher resolution and 3D detection capability, this probe was later combined with fiber Bragg grating [25]. However, the fiber Bragg grating probe is expensive and difficult to manufacture. Stone et al. [26] reported a fiber deflection probing (FDP) method for CMM to inspect the profile of micro cavities. To achieve extremely high optical path magnification in limited measurement space, a fiber probe based on micro focal-length collimation, in which an optical fiber stylus acts as a micro focal-length cylindrical lens, was proposed in 2010 [27]. The method was then improved to include 3D measurement capability [28,29]. The probing system is capable of decoupling 3D tactility, but the devices are too complicated to set up. The setup and calibration of the optical path requires high precision.

Many probing systems perform satisfactorily in probing experiments, but most of them have complicated structures or detection systems. To address this issue, an ACF probe is proposed in this paper. The ACF probe has high detection performance and is compact, inexpensive, easy to fabricate, and has a high signal to noise ratio (SNR) compared to the other probes. 

## 2. Principle

**Figure 1:**
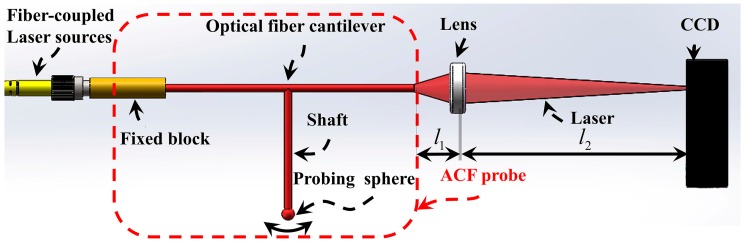
Structure and measurement principle of the assembled cantilever fiber (ACF) probe.

The structure of the ACF touch trigger probe is shown in Figure 1. The ACF probe consists of a fixed block, an optical fiber cantilever, a shaft, and a probing sphere. The shaft is glued to an optical fiber cantilever. The probing sphere is located at the free end of the shaft, which can be glued to the shaft or directly fabricated on the shaft through melting. The advantage of the assembled cantilever structure of the probe is that its detection optical path is located above the objects to be measured, and the structure of the objects has little effect on the measurement result. Accordingly, the probe system can measure high-aspect-ratio microstructures.

The ACF touch trigger probe optical path is simple. The laser beam is coupled into the optical fiber cantilever and transmitted from the fiber cantilever. The transmitted beam converges on the photosensitive surface of the charge couple device (CCD) camera by lens. The free end of the fiber cantilever generates a displacement when the probing sphere touches the object to be measured. Therefore, the deflections of the shaft and probing sphere are transformed into the displacement of the centroid of the light spot, which can be measured by the CCD camera. The detection method can be applied to measure microstructures without complicated operation and its signal to noise ratio (SNR) is high. The transfer function of the optical path can be expressed as:

(1) $$S_{\mathrm{CCD}}=\beta_{\mathrm{op}}\cdot S_{\mathrm{free}}/c_{p},$$

where, $S_{\mathrm{CCD}}$ is the centroid position displacement of the light spot image of CCD, $S_{\mathrm{free}}$ is the displacement of the free end of the fiber cantilever, and $\beta_{\mathrm{op}}$ is the magnifying transfer coefficient of the optical path, which can be expressed as $\beta_{\mathrm{op}}=l_{2}/l_{1}$. Therein, $l_{1}$ is the distance of the free end of the cantilever to the lens, and $l_{2}$ is the distance of the lens to CCD camera photosensitive. $c_{p}$ is the pixel size of the CCD camera. Here, $c_{p}$ is 3.75 μm/pixel.

For the ACF probe system to have high displacement sensitivity, it requires a large magnifying transfer coefficient. However, the increase in the magnifying transfer coefficient results in the volume enlargement of the measuring system. To achieve high displacement sensitivity with a size limitation, the lens with a micro focal length should be applied in the ACF probe system. 

## 3. Analysis and Design of the Probe

### 3.1. Displacement Transfer Function of the Probe

For the ACF probe, different directions of contact force result in different deflections of the probing sphere and fiber cantilever. Therefore, analyzing the effect of the 3D contact force acting on the probing sphere of the shaft is essential.

**Figure 2:**
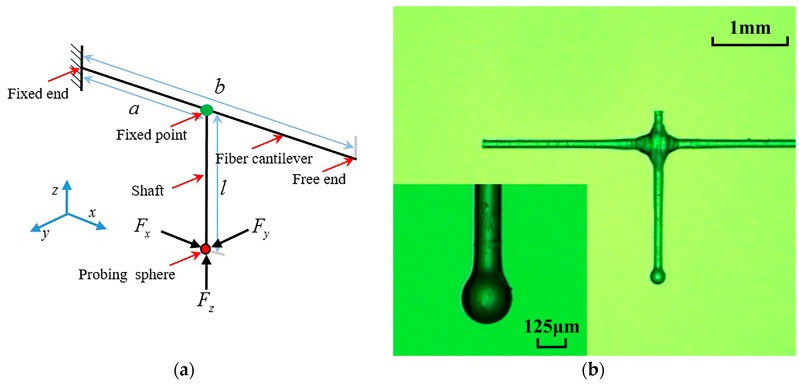
Structure of the probe: (**a**) structure diagram of the ACF probe; and (**b**) photograph of the ACF probe.

The structure diagram and photo of the probe are shown in Figure 2, where *a* is the distance between the shaft and the fixed end of the fiber cantilever, *b* is the length of the fiber cantilever, and *l* is the length of the shaft. As shown in Figure 2a, the contact force along the three orthogonal directions of x, y, and z are loaded on the probing sphere, and any other direction of the contact force can be synthesized with these three directions.

**Figure 3:**
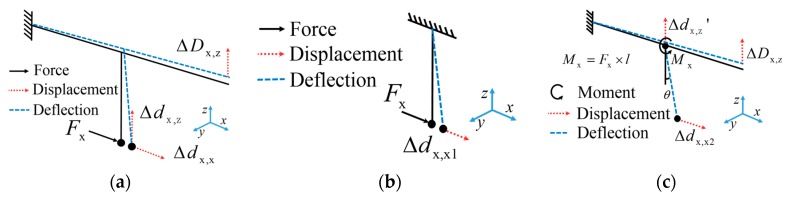
The ACF probe was split and analyzed separately: (**a**) the contact force along the x-axis subject to probing sphere; (**b**) the deflection of the shaft; and (**c**) the deflection of the cantilever.

To determine the relationship between the displacement of the probe and the contact force, the ACF probe was divided into two parts for analysis: one part is the shaft (Figure 3b), and the other is the cantilever beam (Figure 3c). In Figure 3, the solid arrows represent the direction of the force, the dashed arrows indicate the direction of the displacement, the curve arrows represent the direction of the moment, and the broken line indicates the deflection of the probe.

The relationship between displacement and force in the x direction was analyzed. For the shaft of the ACF probe, it can be treated as a traditional single-ended fixed fiber cantilever beam and the force in the x direction is subject to the free end of the shaft (Figure 3b). The displacement of the probing sphere $\Delta d_{x,x1}$ can be obtained by applying deflection equation of cantilever beam:

(2) $$\Delta d_{x,x1}=\frac{F_{x}l^{3}}{3EI},$$

where E and I are the Young’s modules and polar moment of the inertia of the fiber, respectively. The polar moment of the inertia of the fiber is $I=\pi d^{4}/64$, where d is the fiber diameter.

For the cantilever of the ACF probe, it can also be treated as a traditional single-ended fixed fiber cantilever beam while the moment $M_{x}$ is subjected to the free end. $M_{x}$ can be expressed as $M_{x}=F_{x}\cdot l$. The moment $M_{x}$ is located on the fixed point between the shaft and the cantilever. As shown in Figure 3c, the moment leads to a displacement $\Delta D_{x,z}$ of the free end of the cantilever and thus leads to a rotation θ and a displacement Δdx,z' of the shaft along the z-axis. By applying deflection equation of cantilever, the displacement $\Delta D_{x,z}$, Δdx,z', and rotation θ can be expressed as:

(3) Δdx,z'=Fxla22EI,

(4) $$\Delta D_{x,z}=\frac{F_{x}la}{EI}(b-\frac{a}{2}),$$

(5) $$\theta =\frac{F_{x}la}{EI}.$$

The rotation of the shaft will lead to a displacement along the x-axis of the probing sphere:

(6) $$\Delta d_{x,x2}=\theta \times l=\frac{F_{x}l^{2}a}{EI}.$$

The total displacement of the probing sphere consists of two parts. One is obtained by analyzing the shaft, the other is obtained by analyzing the cantilever. The total displacement of the probing sphere and the free end of the cantilever can be expressed as:

(7)  {ΔDx,x=0ΔDx,y=0ΔDx,z=ΔDx,z{Δdx,x=Δdx,x1+Δdx,x2Δdx,y= 0Δdx,z=Δdx,z'.

According to Equations (2) and (7), the relationship between the displacement and force along the x-axis can be expressed as:

(8) $$\{\begin{array}{l} \Delta D_{x,x}=0\\ \Delta D_{x,y}=0\\ \Delta D_{x,z}=\frac{F_{x}la}{EI}(b-\frac{a}{2}) \end{array}\{\begin{array}{l} \Delta d_{x,x}=\frac{F_{x}l^{3}}{3EI}+\frac{F_{x}l^{2}a}{EI}\\ \Delta d_{x,y}=0\\ \Delta d_{x,z}=\frac{F_{x}la^{2}}{2EI} \end{array},$$

where $\Delta D_{i,j}$ is the displacement of the free end of the cantilever along axis j (j = x, y, or z) in the situation of force being applied to the probing sphere along axis i (i = x, y, or z). $\Delta d_{i,j}$ is the displacement of the probing sphere along axis j (j = x, y or z) when force is loaded on the probing sphere along axis i (i = x, y, or z).

The derivation method can also be applied to analyze the displacement of the probing sphere and free end of the cantilever when force is loaded on probing sphere along the y- or z-axis. The relationship between the displacement and the contact along the y-axis can be expressed as:

(9) $$\{\begin{array}{l} \Delta D_{y,x}=0\\ \Delta D_{y,y}=\frac{F_{y}a^{2}}{6EI}(3b-a)\\ \Delta D_{y,z}=0 \end{array}\{\begin{array}{l} \Delta d_{y,x}=0\\ \Delta d_{y,y}=\frac{F_{y}a^{3}}{3EI}+\frac{F_{y}l^{3}}{3EI}+\frac{F_{y}l^{2}a(1+\upsilon )}{EI}\\ \Delta d_{y,z}=0 \end{array}.$$

The relationship between the displacement and the contact force along the z-axis can be expressed as:

(10) $$\{\begin{array}{l} \Delta D_{z,x}=0\\ \Delta D_{z,y}=0\\ \Delta D_{z,z}=\frac{F_{z}a^{2}}{6EI}(3b-a) \end{array}\{\begin{array}{l} \Delta d_{z,x}=0\\ \Delta d_{z,y}=0\\ \Delta d_{z,z}=\frac{F_{z}a^{3}}{3EI} \end{array}.$$

In reality, the contact force can be expressed as a stack of these three directions. Therefore, according to Equations (8)–(10), the relationship function between the probing sphere and contact force can be expressed as Equation (11), and the relationship function between the displacement of the free end of the cantilever and contact force can be expressed as Equation (12).

(11) $$\left[\begin{array}{l} \Delta d_{x}\\ \Delta d_{y}\\ \Delta d_{z} \end{array}\right]=\frac{1}{6EI}\left[\begin{array}{ccc} 2l^{3}+6l^{2}a & 0 & 0\\ 0 & 2a^{3}+2l^{3}+6l^{2}a(1+v) & 0\\ 3a^{2}l & 0 & 2a^{3} \end{array}\right]\left[\begin{array}{l} F_{x}\\ F_{y}\\ F_{z} \end{array}\right],$$

(12) $$\left[\begin{array}{l} \;0\\ \Delta D_{y}\\ \Delta D_{z} \end{array}\right]=\frac{1}{6EI}\left[\begin{array}{ccc} 0 & 0 & 0\\ 0 & a^{2}(3b-a) & 0\\ al(6b-3a) & 0 & a^{2}(3b-a) \end{array}\right]\left[\begin{array}{l} F_{x}\\ F_{y}\\ F_{z} \end{array}\right],$$

where ${\left[\Delta d_{x}\;\Delta d_{y}\;\Delta d_{z}\right]}^{T}$ is the displacement of the probing sphere, ${\left[0\;\Delta D_{y}\;\Delta D_{z}\right]}^{T}$ is the displacement of the free end of the fiber cantilever, ${\left[F_{x}\;F_{y}\;F_{z}\right]}^{T}$ is the contact force along the three axes to which the probing sphere is subjected, and v is the Poisson ratio of the fiber.

Because the deflection of the fiber cantilever along the axis x is small, we considered that the displacement of the free end of the cantilever only exists in the y-z plane, and thus, the displacement of the free end of the fiber cantilever can be expressed as ${\left[0\;\Delta D_{y}\;\Delta D_{z}\right]}^{T}$. The displacement of the probe tip in the x, y, and z directions transforms into the displacement of the free end of the fiber cantilever in the y and z directions. The detection system makes it impossible to distinguish between a displacement along the x axis or the z axis, meaning it can only be used as a touch trigger probe. However, the ACF probe can sense the force from three directions and can be used for 3D measurement. In most cases, the basic profiles of the samples are already known, according to the blueprints or observation. So, the contact directions can be predicted when the ACF probe, combined with a 3D positioning stage, is applied to measure the objects. Although the trigger signal cannot distinguish the contact direction, it has little influence when measuring objects with clear structures.

From Equations (11) and (12), the displacement transfer function between the probing sphere and the free end of the cantilever can be expressed as:

(13) $$\left[\begin{array}{l} 0\\ \Delta D_{y}\\ \Delta D_{z} \end{array}\right]=\left[\begin{array}{ccc} 0 & 0 & 0\\ 0 & \frac{3a^{2}b-a^{3}}{2a^{3}+2l^{3}+6l^{2}a(1+v)} & 0\\ \frac{3a(b-a)}{4l^{2}+12la} & 0 & 1.5b/a-0.5 \end{array}\right]\left[\begin{array}{l} \Delta d_{x}\\ \Delta d_{y}\\ \Delta d_{z} \end{array}\right].$$

From Equation (13), the displacement transfer function is only related to the structure parameters of the probe.

### 3.2. Finite Element Analysis of the Probe

A finite element analysis for the probe has also been performed to verify the correctness of the Equation (13). The parameters listed in Table 1 have been used, where, the theoretical transfer coefficients are calculated by substituting the structure parameters v, b, a and l into the Equations (11) and (12).

The contact force is applied to the probing sphere in the x, y and z directions. The displacement of the probing sphere and the free end of the fiber cantilever is calculated by finite element simulation, therefore, the simulation displacement transfer function between the probing sphere and the free end of the cantilever can be expressed as:

(14) $$\left[\begin{array}{l} 0\\ \Delta D_{y}\\ \Delta D_{z} \end{array}\right]=\left[\;\begin{array}{ccc} 0 & 0 & 0\\ 0 & 0.4568 & 0\\ 0.1879 & 0 & 2.502 \end{array}\right]\left[\begin{array}{l} \Delta d_{x}\\ \Delta d_{y}\\ \Delta d_{z} \end{array}\right].$$

Compared with the theoretical transfer coefficient in Table 1, it can be found that the simulation results are very close to the theoretical calculation. Thus, the correctness of Equation (13) can been verified. The reason for the small discrepancy between the simulation and the theoretical solution is that the contact point is on the sphere surface and not in the middle of the fiber in the simulation. 

**Figure 4:**
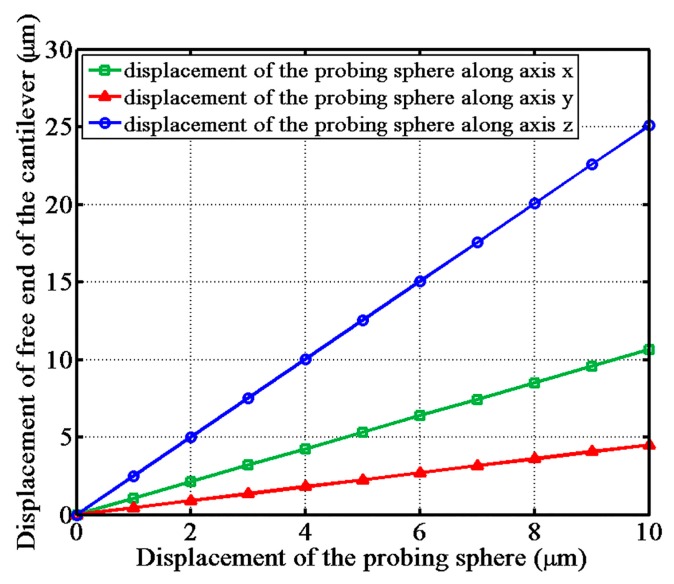
The displacement relationship of the probing sphere and the free end of the fiber cantilever obtained by finite element analysis.

By loading a step-increasing displacement to the probing sphere, the displacement relationship between the probing sphere and the free end of the cantilever is shown in Figure 4. It is obvious that the transfer coefficient along the axis z is the highest, and the transfer coefficient along axis y is the lowest. It also indicates that the detection sensitivity is highest in the z direction and the lowest in the y direction.

### 3.3. Optimal Design of the Probe

To obtain high detection sensitivity, the ACF probe structure was optimized. The effects of the structure parameters were analyzed, mainly including the distance between the shaft and the fixed end of the cantilever (*a*), the length of the cantilever (*b*), and the length of the shaft (*l*).

According to Equation (11), when the contact force is ${\left[F_{x}\;0\;0\right]}^{T}$, the probing sphere has a displacement along axes x and z, the displacement of the two axes can be expressed as $\Delta d_{x,x}=(2l^{3}+6l^{2}a)F_{x}$ and $\Delta d_{x,z}=3a^{2}lF_{x}$, respectively, and their relationship can be expressed as:

(15) $$\Delta d_{x,z}=\frac{3a^{2}}{2l^{2}+6la}\Delta d_{x}.$$

**Figure 5:**
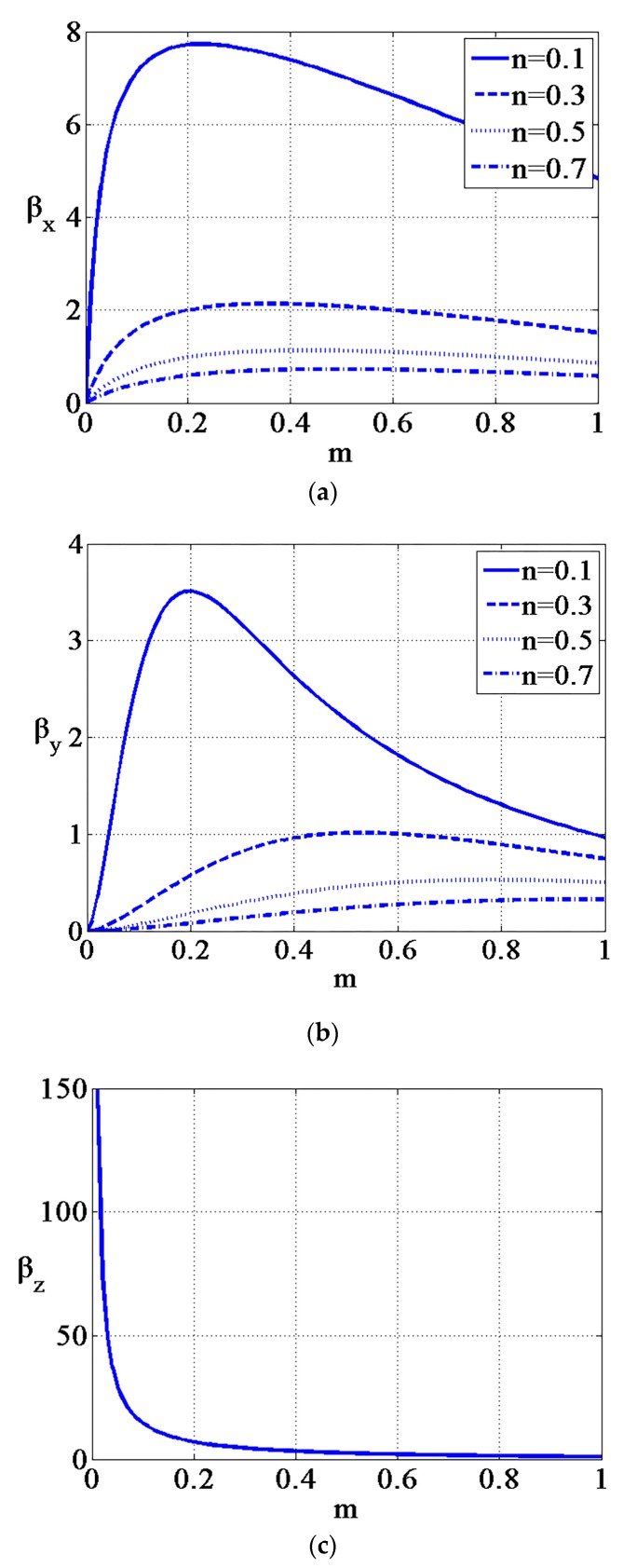
The relationship between parameters *m*, *n*, and the transfer coefficient: (**a**) the curve of the x-axis transfer coefficient; (**b**) the curve of the y-axis transfer coefficient; and (**c**) the curve of the z-axis transfer coefficient.

According to Equations (1), (13) and (15), the total displacement transfer function and transfer coefficients can be expressed as:

(16) $$\left\{ \begin{array}{l} \Delta S_{x,z}=\beta_{\mathrm{op}}\beta_{x}\Delta d_{x}/c_{p}\\ \Delta S_{y,y}=\beta_{\mathrm{op}}\beta_{y}\Delta d_{y}/c_{p}\\ \Delta S_{z,z}=\beta_{\mathrm{op}}\beta_{z}\Delta d_{z}/c_{p} \end{array}\right.,$$

(17) $$\left\{ \begin{array}{l} \beta_{x}=\frac{3m\left(2-m\right)}{2n^{2}+6nm}\\ \beta_{y}=\frac{m^{2}\left(3-m\right)}{2m^{3}+2n^{3}+6mn^{2}(1+v)}\\ \beta_{z}=\frac{3/m-1}{2} \end{array}\right.,$$

where $\Delta S_{i,j}$ the centroid position displacement of the light spot image of the CCD along axis j (j = x or z), when the force is loaded on the probing sphere along axis i (i = x, y, or z); $\beta_{i}$ is the transfer coefficient along axis i (i = x, y or z); *m* is the relative position of the shaft fixing point; and *n* is the relative length of the shaft, and they can be expressed as *m* = *a*/*b* and *n* = *l/b*. The two parameters *m* and *n* are quite important, as they determine the sensitivity of the probe. The relationship curve between transfer coefficient $\beta_{i}$ and *m* and *n* are shown in Figure 5.

Figure 5a,b show that a small value of *n* results in high radial sensitivity of the ACF probe. However, the value of *n* cannot be set as small as possible. To ensure the depth of the microstructures can be detected, the value of *l* is determined. Considering that n=l/b, the value of *b* will increase with the decrease in *n*, which decreases the natural frequency of the ACF probing. Figure 5a,b also indicate that optimal solutions exist for $\beta_{x}$ and $\beta_{y}$ in the interval of *m* from 0 to 1. The appropriate value of *m* should be selected according to the measurement process. If the measurement process requires high sensitivity along the x-axis, the corresponding values of *m* and *n* should be selected according to the relationship curve in Figure 5a. Figure 5c indicates that the axial transfer coefficient $\beta_{z}$ is approximately inversely proportional to the value of *m*, so that a decrease in the value of *m* leads to an increase in axial detection sensitivity.

Another important parameter of the ACF probe is the diameter of the probing sphere that determines the measurable diameter of the micro-hole. A smaller sphere diameter can be obtained by etching the fiber to expand the measuring range.

## 4. Experiment

### 4.1. Fabrication of the ACF Probe

The single mode fiber with an operating wavelength of 532 nm was used to fabricate the ACF probe. A melting probing sphere was fabricated using electric discharge machining (AV6471A, The 41st Institute of China Electronics Technology Group Corporation, Qingdao, China) at the end of the fiber whose coating layer had been stripped. The optical fiber, with its end cut flat, was placed between the discharge electrodes. The high voltage discharges generated by the electrodes break down the air to create a high temperature arc, and the instantaneous high temperature melted the end of the fiber. The melted fiber ends condensed into a sphere under surface tension. The process factors during the fabrication of the probe sphere were as follows: pre-melting time of 85 ms, melting time of 85 ms, pre-melting current of 40 mA, and melting current of 40 mA. Then, the fiber with the sphere was cut to a length of 5 mm using a fiber cleaver as the shaft. 

The shaft was assembled vertically to the cantilever using cyanoacrylate adhesive. The distance between the fixed point and the free end of the cantilever was 5 mm. Assembly operation was performed under a video measuring instrument. After the glue was completely cured, the probe was carefully fixed on the mechanical part, and the distance between the fixing point and the free end of the cantilever was about 10 mm. The last step was to fabricate the fiber patch cables on the other end of the cantilever to connect the fiber laser, finishing the fabrication of the ACF probe. The length of parameters *b*, *a*, and *l* of the probe were 10 mm, 5 mm, and 5 mm, respectively, and thus *m* = 0.5 and *n* = 0.5.

**Figure 6:**
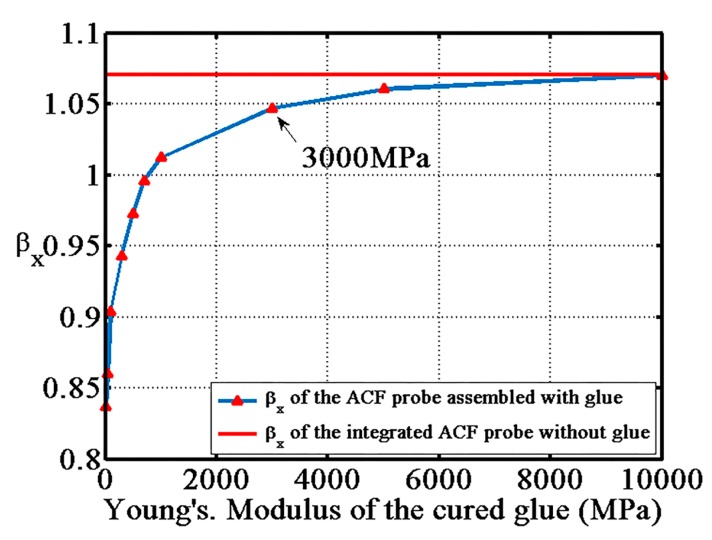
The relationship between the Young’s modulus of the cured glue.

The glue used in the fabrication process of the ACF probe was chosen carefully because the elasticity of the glue influences the displacement of the shaft. In Figure 6, the blue line illustrates the influence of Young’s modulus of the curved glue on the x-axis transfer coefficient, and other two axes have similar curves. The red line indicates the transfer coefficient of ideal structure of the ACF probe. The ideal structure refers to the cantilever beam and the shaft being integrated without glue. The parameters listed in Table 1 were used for finite element analysis. By loading the same displacement along the x-axis to the probing sphere of the probe with different glues, the transfer coefficient was calculated using finite element simulation. From Figure 6, when the Young’s modulus of the cured glue is larger than 3000 MPa, the x-axis transfer coefficient is stable and is close to the coefficient of the probe with the ideal structure. The cyanoacrylate adhesive (V-100, Tong Shen Enterprise Co., Ltd., Kaohsiung, Taiwan) that was used in experiments has a Young’s modulus between 3000 and 5000 MPa. Assuming that the Young’s modulus of the glue is 3000 MPa, the x-axis transfer coefficient of the probe is 1.04 by finite element analysis, while the x-axis transfer coefficient is 1.07. Because the value of the two coefficients are close, the cyanoacrylate adhesive was used to glue the shaft and the cantilever. 

### 4.2. Experimental Setup

**Figure 7:**
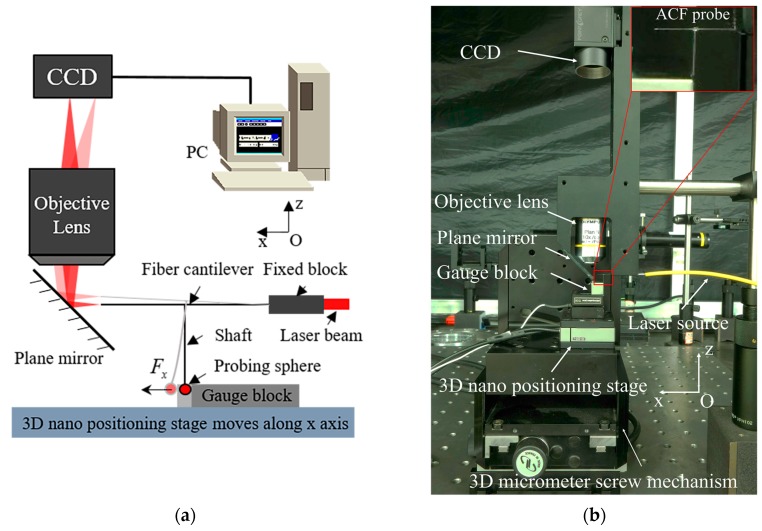
Experimental setup of the ACF probing system: (**a**) experimental schematic diagram of the probing system; and (**b**) experimental setup of the ACF probing system.

The ACF probe system was set up. All experimental equipment were placed on the vibration isolation platform. A glass cover was used to reduce the influence of air. The wavelength of the fiber-couple laser source was 532 nm. The CCD camera with 1288 × 964 pixels and pixel size of 3.75 μm (FL3-GE-13S2M-C, FLIR Inc., Wilsonville, OR, USA) were used to detect the shift in the light spot. The mechanical structure was designed to be used in conjunction with the ACF probe for measurement. The objective lens with an effective focal length (EFL) of 18 mm and numerical aperture (NA) of 0.25 (RMS10X, Olympus Corporation, Tokyo, Japan) was screwed into the mechanical structure, and could obtain an optical magnifying coefficient $\beta_{\mathrm{op}}$ of around 11. A micro displacement stage was constructed, in which large range coarse positioning was achieved with a 3D micrometer screw mechanism, and nanometer positioning was achieved with a 3D piezoelectric nano positioning stage, assembled using a z nano positioning stage (PZ-38, piezosystem jena GmbH, Jena, Germany) and x-y nano positioning stages (PXY-100, piezosystem jena GmbH, Jena, Germany). The z stage was stacked onto the x-y stage. The precision gauge block was fixed onto the nano positioning stage to touch the ACF probe. The experimental set-up was configured as shown in Figure 7.

### 4.3. Probe Performance Experiment

**Figure 8:**
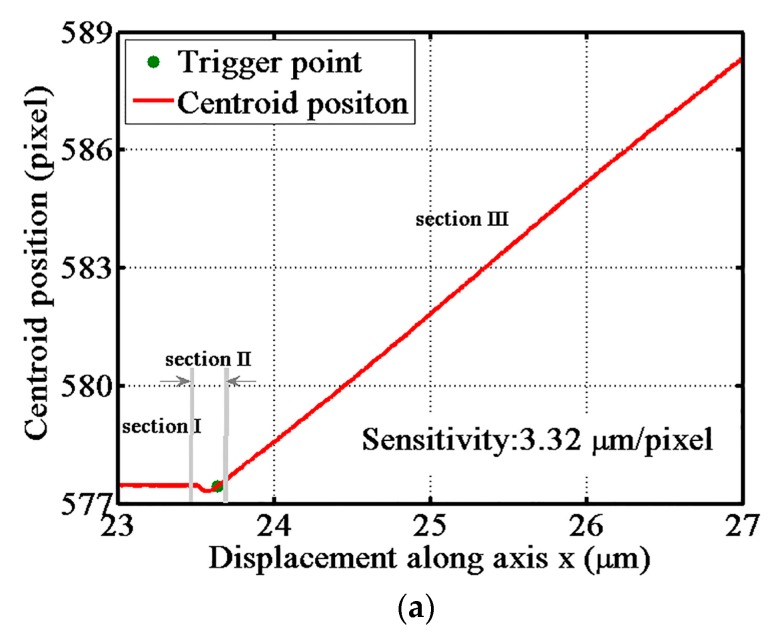

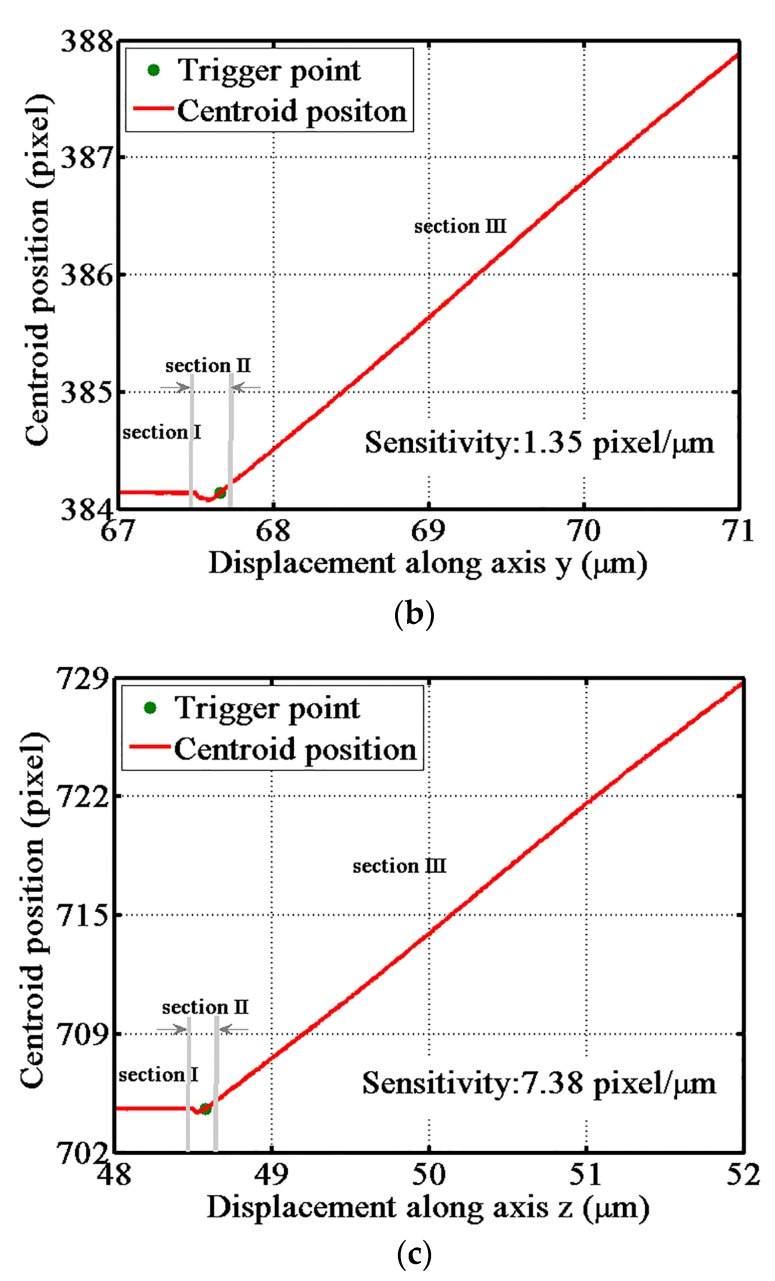
Output response curves of the probe: (**a**) Gauge block moves along the x-axis; (**b**) Gauge block moves along the y-axis; and (**c**) Gauge block moves along the z-axis.

Experiments were performed to evaluate the performance of the ACF fiber probe. The precision gauge block moves along three directions to touch the probing sphere. The output response curves of the probe along the x, y, and z axes are shown in Figure 8. The sampling frequency of the CCD camera was one frame per 10 nm. The size of the light spot on the CCD camera was more than 200 × 200 pixels and the spot shape was round with good quality. The center-of-gravity algorithm was applied to calculate the position of the light spot centroid and a 0.01-pixel resolution of the CCD camera output was achieved. In Figure 8, section I shows when the block has not yet touched the probing sphere. At this time, the ACF probe is in the non-triggered state, and the centroid position of the light spot remains constant. In section II, the outputs of the CCD camera change suddenly, caused by the interfacial adhesive force. The interfacial adhesive force resulted from the co-action of the van der Waals force, capillary force, and other micro-forces [9]. In section III, the centroid position of the light spot changed linearly with the increasing displacement of the nano positioning stage. Now, the probe was in the over-triggered state. The detection sensitivity was determined by the curve slope of the curve fitted line in section III. The detection sensitivity of the probe along the x-axis was 3.32 pixels/μm, and the sensitivity of the probe along the y and z axes were 1.35 pixels/μm and 7.38 pixels/μm, respectively. What is more, the probe has a long linear trigger range more than 4 μm that is large enough for the touch trigger measurement. The sensitivities of the probe were highest in the z direction and the lowest in the y direction, corresponding to the displacement relationship curves shown in Figure 4. 

The position of the touch trigger point is so important that it should be determined as accurately as possible. By adopting the linear fitting method, the effect of the position vibration of the touch trigger point was eliminated. The centroid position of the non-triggered state and the centroid position of the over-triggered were fitted to two straight lines, and the intersection of the two straight lines was seen as the touch trigger point. As shown in Figure 8, the green solid circle is the trigger point calculated using the linear fitting method.

**Figure 9:**
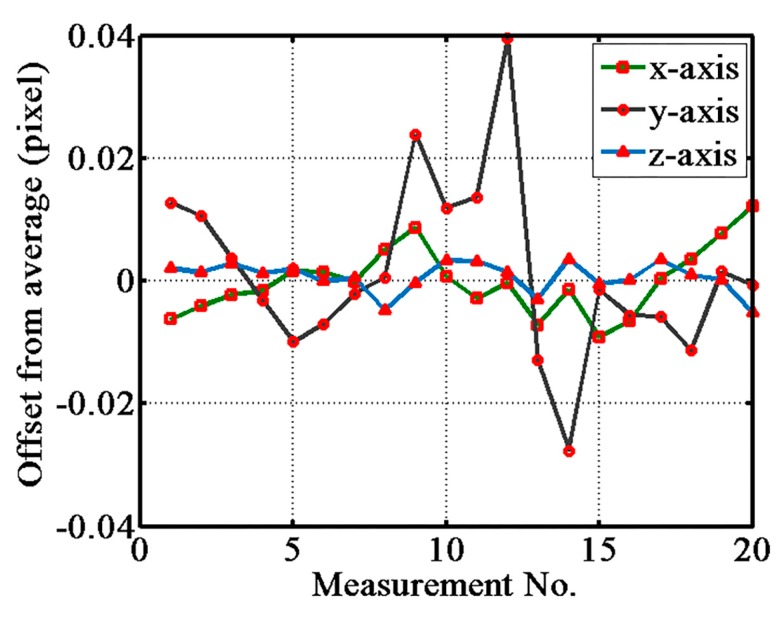
Repeatability results along the x, y, and z axes.

The repeatability of the ACF probe along the x, y, and z axes was also tested. At the beginning of this experiment, the gauge block was controlled to move to the non-triggered state, corresponding to section I in Figure 8, and then was moved along each direction to touch the probe repeatedly with a displacement of 2 μm. Figure 9 shows the repeatability results of 20 repetitions along the x, y, and z axes. The vertical axis indicates the offsets from the average displacement value of the trigger point. The standard deviations of the x, y, and z directions were 20.6 nm, 54.4 nm, and 9.4 nm, respectively.

**Figure 10:**
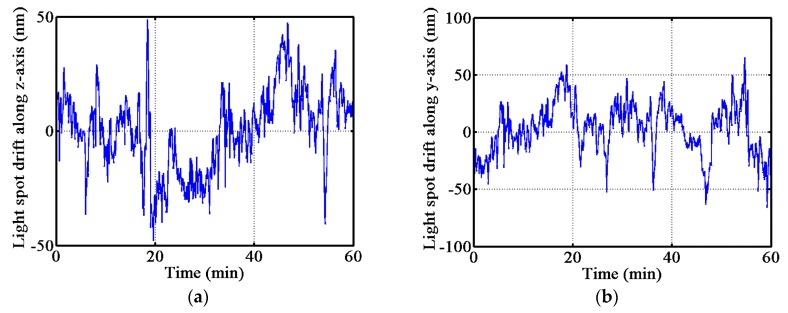
Stability experiment result of the probe system. (**a**) The drift of light spot along the z-axis, and (**b**) the drift of the light spot along the y-axis.

The stability of the probe system in the y and z axes was tested. The Figure 10 shows the stability test results of the probe. The total drift of the light spot in the y and z axes were about 130 nm and 100 nm for a duration of one hour, respectively. The drift of the light spot was mainly caused by environmental factors such as vibration of the experiment platform, air disturbances, and temperature changes. Observing the probe along the x, y, and z axes, the equivalent area in the y-axis was the largest. So, the ACF probe in the y-axis is more sensitive to external forces, and especially to air disturbances, than the other two axes, which is why the y-axis stability of the ACF probe is larger than the z-axis.

**Figure 11:**
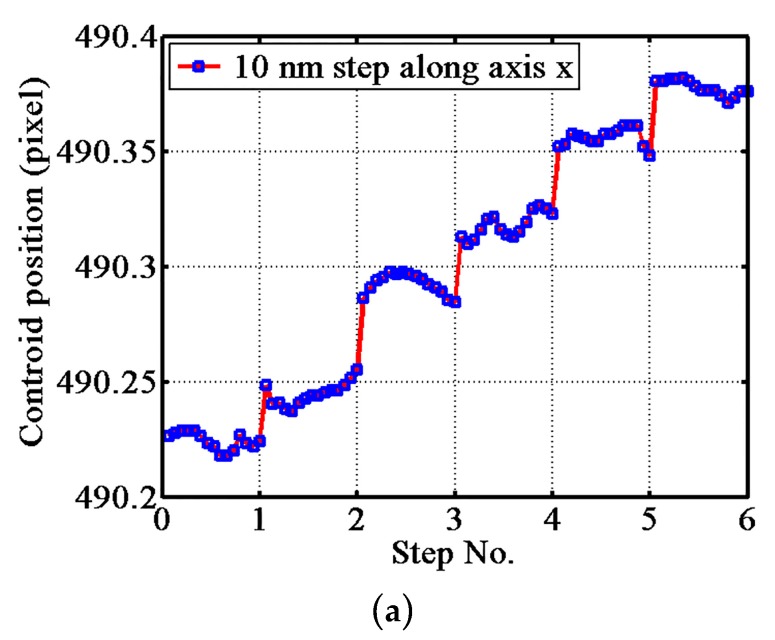

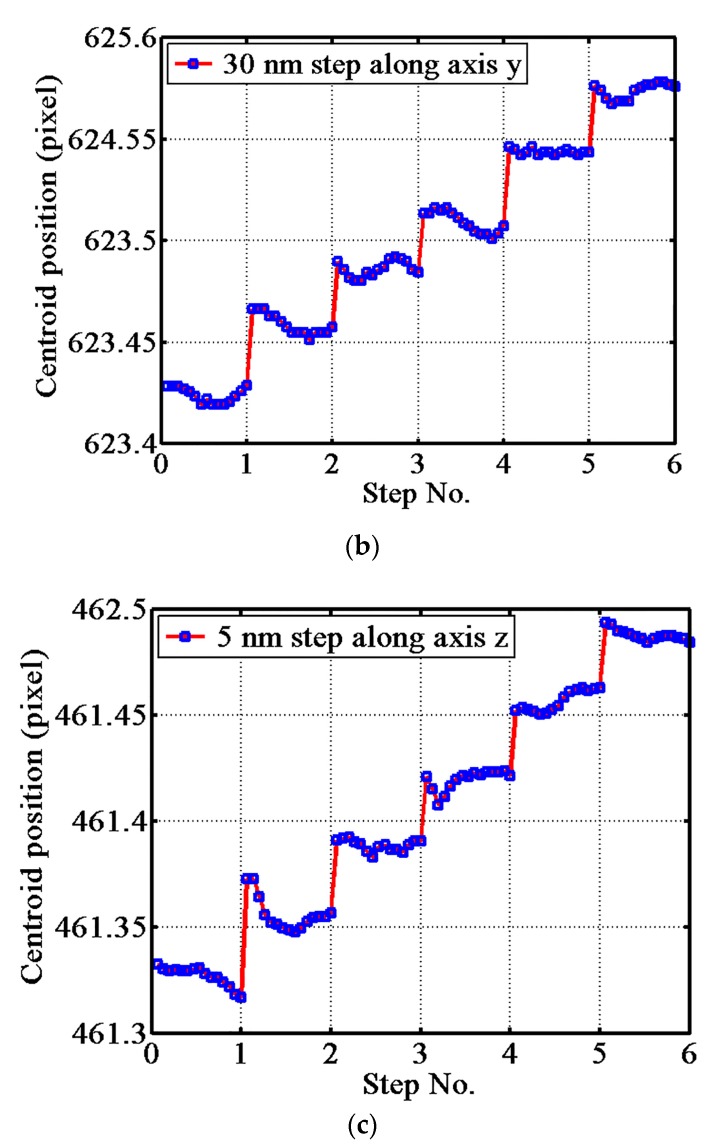
Resolution of the ACF probe: (**a**) along the x-axis, (**b**) along the y-axis; and (**c**) along the z-axis.

An experiment using the same conditions as the repeatability test was completed to investigate the resolution of the probe, and the results are shown in Figure 11. At the beginning of this experiment, the gauge block was controlled to move it to linear response section, corresponding to section III in Figure 8, and then was moved in regular steps. The horizontal axis of Figure 11 is the number of steps of the nano positioning stage and the vertical axis is the shift of the CCD spot centroid. The minimum moving step that the probe system could resolve is the displacement resolution in this direction. The resolution of the ACF probing system along axes x, y, and z were 10 nm, 30 nm, and 5 nm, respectively.

### 4.4. Calibration Experiments and Uncertainty Analyses

**Figure 12:**
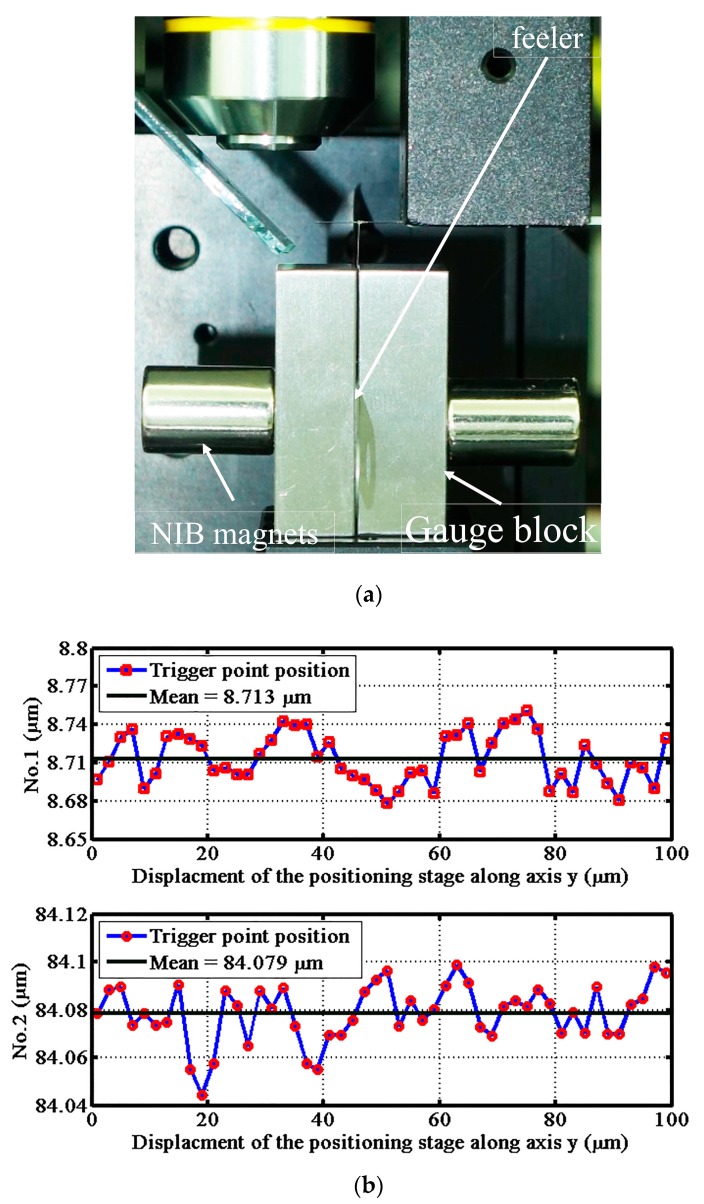
Measurement of the silt: (**a**) photograph of the micro slit and (**b**) the measurement results of the two trigger points.

There were two purposes to the micro-slit experiment: to verify the high aspect ratio measurement capability of the ACF probe and to calibrate the diameter of the probing sphere [30]. The diameter of probing sphere, measured by the video measurement system, was 174.4 ± 2.8 μm. Poor sphere-diameter accuracy makes performing high precision measurements impossible. So, the diameter of the sphere had to be calibrated. The coordinates of the ACF probe, gauge blocks, and nano positioning stage were carefully aligned to reduce the alignment error. A precision feeler gauge, with a thickness of 250 μm grade K referred to as a calibrated gauge, was placed in the middle of the two support gauge blocks with grade K to achieve a micro slit to qualify the probe system. As shown in Figure 12a, neodymium iron boron (Nd_2_Fe_14_B) magnets were used to fasten the two gauge blocks. Figure 12b shows the measurement results of the number one and number two trigger point positions. 

To avoid the influence of the edge chamfers of the gauge blocks, the probe sphere was aligned inside the gap with a depth of more than 100 μm from the top surface of the gauge blocks. The micro slit was moved back and forth along the x-axis to touch the probing sphere twice, then was moved along the y-axis in a 2-μm step. The total displacement along the y-axis was 100 μm. This detection method avoids the low resolution drawback of the y-axis. The mean trigger point position of the number one position was 8.713 μm, and that of the number two position was 84.079 μm. The mean value and the standard deviation of the measured micro silt were 75.366 μm and 0.023 μm, respectively. The standard deviation of the measurement result was mainly caused by the interfacial adhesive force and the repeatability of the x-y nano positioning stage. According to the measurement result, the mean value of the effective two-point probe sphere diameter was evaluated at 174.634 μm with a standard deviation of 0.023 μm. 

Uncertainty analyses were completed according to GUM (ISO Guide to the Expression of Uncertainty in Measurement). The 0.023-μm standard deviation of measurement result is one of the sources of uncertainty. The corresponding uncertainty was estimated to be 0.014 μm. In addition, the length deviation limit of the calibrated feeler gauge and the supporting gauge blocks were 50 nm. So, the uncertainty of both the calibrated feeler gauge and the supporting gauges block were estimated to be 0.029 μm. The wringing error between the two gauge blocks and feeler gauge had to be considered. A wringing error of 10 nm occurred on each side, according to the data sheet of the gauge block, contributing to an uncertainty of 0.012 μm. The cosine error, Abbe error, and alignment error could also be uncertainty sources in the experiment. However, they are relatively small in the experiment and have little influence on the combined uncertainly. Therefore, the combined uncertainty of the measurement was estimated to be 0.045 μm. Notably, the primary motivation of this paper was to propose a novel probe system. Detailed discussion and improvement of the measurement uncertainty will be completed in future work.

## 5. Conclusions

An assembled cantilever optical fiber touch trigger probe for 3D measurement of microstructures was developed, fabricated, and tested. The experimental results showed that the ACF probe has the potential to achieve nanoscale measurement resolution in 3D measurements of microstructures. The probe has the powerful capability to meet the 3D precision measurement requirements. Furthermore, the ACF probe system has many advantages, such as being inexpensive, easy to fabricate, compact, with high SNR. In the future, a lens with a micro focal length will be used for ultra-high sensitivities. 

## Figures and Tables

**Table 1:** The parameters of the probe.

Parameter	Value
E Young’s modulus of the optical fiber (Gpa)	80
d Diameter of the optical fiber (μm)	125
b (mm)	4
a (mm)	2
l (mm)	2
Diameter of the probing sphere (μm)	150
Contact force (μN)	20
v Poisson ratio of the fiber	0.16
Theoretical transfer coefficient (∆*D*_z_/∆*d*_x_)	0.1875
Theoretical transfer coefficient (∆*D*_y_/∆*d*_y_)	0.4562
Theoretical transfer coefficient (∆*D*_z_/∆*d*_z_)	2.500
